# Increased Rate of Epigenetic Aging in Men Living With HIV Prior to Treatment

**DOI:** 10.3389/fgene.2021.796547

**Published:** 2022-02-28

**Authors:** Mary E. Sehl, Elizabeth Crabb Breen, Roger Shih, Larry Chen, Ruibin Wang, Steve Horvath, Jay H. Bream, Priya Duggal, Jeremy Martinson, Steven M. Wolinsky, Otoniel Martinez-Maza, Christina M. Ramirez, Beth D. Jamieson

**Affiliations:** ^1^ Division of Hematology-Oncology, Department of Medicine, David Geffen School of Medicine at UCLA, University of California, Los Angeles, Los Angeles, CA, United States; ^2^ Cousins Center for Psychoneuroimmunology, Department of Psychiatry and Behavioral Sciences, David Geffen School of Medicine at UCLA, University of California, Los Angeles, Los Angeles, CA, United States; ^3^ UCLA Computational and Systems Biology Interdepartmental Program, University of California, Los Angeles, Los Angeles, CA, United States; ^4^ Department of Epidemiology, Johns Hopkins Bloomberg School of Public Health, Baltimore, MD, United States; ^5^ Department of Human Genetics, David Geffen School of Medicine at UCLA, University of California, Los Angeles, Los Angeles, CA, United States; ^6^ Department of Molecular Microbiology and Immunology, Johns Hopkins Bloomberg School of Public Health, Immunology Training Program, Johns Hopkins School of Medicine, Baltimore, MD, United States; ^7^ Department of Infectious Diseases and Microbiology, Graduate School of Public Health, University of Pittsburgh, Pittsburgh, PA, United States; ^8^ Department of Medicine, Northwestern University Feinberg School of Medicine, Chicago, IL, United States; ^9^ Departments of Obstetrics and Gynecology and Microbiology, Immunology and Molecular Genetics, David Geffen School of Medicine at UCLA, University of California, Los Angeles, Los Angeles, CA, United States; ^10^ Fielding School of Public Health, University of California, Los Angeles, Los Angeles, CA, United States

**Keywords:** HIV, aging, DNA methylation, epigenetic clock, telomeres

## Abstract

**Background:** Epigenetic aging is accelerated in tissues of persons living with HIV (PLWH) and may underlie the early onset of age-related illnesses. This study examines the rate-of-change in epigenetic age in PLWH following HIV infection but before HAART, using archived longitudinal samples from the Multicenter AIDS Cohort Study.

**Methods:** DNA was isolated from cryopreserved peripheral blood mononuclear cells from 101 men living with HIV, with baseline visit <2.5 years after HIV seroconversion (Visit 1) and follow-up visit <1.5 years before the initiation of HAART (Visit 2), and 100 HIV-uninfected men matched on age and visits with comparable time intervals. DNA methylation (DNAm) age was estimated for five clocks (Pan-tissue, Extrinsic, Phenotypic, Grim, and Skin & Blood age), and a DNAm-based estimate of telomere length (DNAmTL). Multivariate linear regression models were used to examine baseline factors associated with rate-of-aging, defined as (DNAm age visit 2–DNAm age visit 1)/(age visit 2–age visit 1).

**Results:** Epigenetic age increased approximately twice as fast in PLWH as uninfected controls (Pan-tissue, Extrinsic, and Phenotypic clocks). Shortening of DNAmTL was nearly 3-fold faster in PLWH than controls. Faster rate-of-aging was associated with HIV status (Pan-Tissue, Extrinsic, Phenotypic, and DNAmTL), white race (Extrinsic, DNAmTL), higher cumulative HIV viral load (Grim), and lower baseline DNAm age (Phenotypic, Skin & Blood).

**Conclusion:** Epigenetic rates-of-aging were significantly faster for untreated PLWH. Our findings expand on the important impact of HIV infection on biologic aging, both in elevating epigenetic age and increasing the rate-of-aging in the years following infection.

## Introduction

Persons living with HIV have an increased risk of age-related chronic illnesses, including dementia, diabetes, cardiovascular disease, chronic lung disease, stroke, osteoporosis, frailty, and some cancers (Schouten et al., 2014; Friedman and Duffus, 2016; Bigna et al., 2018; Gelpi et al., 2018; Mayer et al., 2018; Yang et al., 2019). While body compositional changes, chronic inflammation, and immunosenescence have been demonstrated in the setting of HIV infection (Cao et al., 2009; Brown et al., 2010; Rickabaugh et al., 2011; Koethe et al., 2020), the precise underlying molecular mechanisms driving premature aging and its associated risks remain poorly understood. Biologic aging, as estimated using DNA methylation patterns, is accelerated in whole blood, peripheral blood mononuclear cells, CD4^+^ T lymphocytes and monocytes, and brain tissue of persons living with HIV (Horvath and Levine, 2015; Rickabaugh et al., 2015; Gross et al., 2016). By examining age-related methylation patterns using epigenetic clock and global methylation analyses, the degree of acceleration has been estimated to be around 5–14 years in peripheral blood (Horvath and Levine, 2015; Rickabaugh et al., 2015; Gross et al., 2016) and 7 years in brain tissue (Horvath and Levine, 2015). The effects of HIV on DNA methylation age extend across a wide range of ages, occurring as early as adolescence in children who have acquired HIV infection perinatally (Horvath et al., 2018a; Shiau et al., 2021). Recently, accelerated epigenetic aging has been shown to occur as early as 2–3.3 years after the time of infection (Esteban-Cantos et al., 2021a) and (Breen *submitted/under review*) and persists after highly active anti-retroviral therapy (HAART) is initiated, with only a partial reduction in the degree of acceleration (Sehl et al., 2020; Esteban-Cantos et al., 2021b; Corley et al., 2021).

A recent focus in aging and epigenetics research has been to quantify the pace of epigenetic aging (Snir and Pellegrini, 2018; Farrell et al., 2020), and identify methylation predictors of the rate of change in biomarkers tracking organ system integrity (Belsky et al., 2020). However, relatively few studies have examined rate-of-change in epigenetic age in the setting of HIV infection. No prior extensive studies have examined longitudinal changes of epigenetic age over the years following documented initial HIV infection, with accompanying longitudinal data from an age-matched uninfected control group. Furthermore, at both visits, the HIV-infected participants in our study had not initiated HAART, allowing us to examine the effects of infection alone over time. The Multicenter AIDS Cohort Study is a unique resource to study longitudinal changes in epigenetic changes over time, housing a richly annotated data set of specimens collected longitudinally in men living with HIV and uninfected controls, with specimens collected prior to the HAART era. In this study, we examine rates of epigenetic aging in men living with HIV in the period spanning just after HIV seroconversion (within 2.5 years) to shortly before HAART initiation (<1.5 years prior).

## Methods

### Study Population and Sample Selection

We obtained viably-frozen peripheral blood mononuclear cells (PBMC) from the Multicenter AIDS Cohort Study (MACS), an ongoing prospective study of the natural and treated history of HIV infection in men who have sex with men (Kaslow et al., 1987). The MACS is part of the MACS/WIHS Combined Cohort Study (MWCCS). Study participants provided informed consent for research upon enrolling in the MACS. The current study was granted exemption status by the University of California, Los Angeles, Medical Institutional Review Board (IRB#15001179).

Participants for our current substudy were selected from a large biomarker substudy within the MACS (Wada et al., 2015). We selected 101 HIV seroconverters who had viable PBMC available <2.5 years post-seroconversion (baseline Visit 1), and <1.5 years prior to the first report of initiation of HAART (Visit 2). For 100 of these seroconverters, matched seronegative controls were selected, matched on age (+/− 2 years), and hepatitis C virus (HCV) status (HCV RNA positive or negative at each visit), with available viable PBMC equivalent to post-seroconversion and pre-HAART visits in a given seroconverter. The time interval between Visits 1 and 2 in the seronegative control was required to be comparable to the time interval in the seroconverter case (+/− 2 years) unless that interval was <2 years, when the interval was then matched as closely as possible to the case. One seroconverter could only be matched at the post-seroconversion visit within 2.3 years of age of the selected seronegative control due to HCV status. Another seroconverter could not be matched to any MACS HIV seronegative controls within the required PBMC, age, and HCV matching criteria.

### DNA Extraction and Methylation Arrays

PBMC were thawed and assayed for viability using trypan blue; the mean viability of PBMC was 89.6%. A total of 1 × 10^6^ viable PBMC were used for genomic DNA isolation using QIAGEN DNeasy mini spin columns (QIAGEN, Germantown, MD). The quality of DNA samples was assessed using Nanodrop measurements, and DNA concentrations were determined using a Qubit assay kit (Life Technology, Carlsbad, CA). 500 ng of DNA was bisulfate-converted using the EZ-methylation kit (Zymo Research) and hybridized to the Infinium HumanMethylation EPIC (850K) array (Illumina, San Diego, CA). Fluorescence data from the hybridized chip were scanned on an iScan (Illumina) and analyzed. Each chip contained a balanced selection of samples drawn from each group (case and control, Visit 1, and Visit 2). DNA methylation levels (β-values) were determined by calculating the ratio of intensities between methylated (signal A) and un-methylated (signal B) sites. We used the “noob” normalization method implemented in the minfi R package (Triche et al., 2013; Fortin et al., 2017). Specifically, the β value was calculated from the intensity of the methylated (M corresponding to signal A) and un-methylated (U corresponding to signal B) sites, as the ratio of fluorescent signals beta = Max (M, 0)/[Max (M, 0) + Max (U, 0) + 100]. Thus β values range from 0 (completely un-methylated) to 1 (completely methylated).

### Epigenetic Clocks and Estimate of Telomere Length

We examined five measures of epigenetic age, estimated from weighted elastic net regression models using methylation levels at selected CpGs from our Infinium MethylationEPIC BeadChip data. These measures include Horvath’s Pan-tissue age, based on 353 CpGs (Horvath, 2013), Extrinsic age, based on 71 CpGs (Hannum et al., 2013), Phenotypic Age, based on 513 CpGs (Levine et al., 2018), Grim Age, based on 1,030 CpGs (Lu et al., 2019a), and Skin & Blood Age, based on 391 CpGs (Horvath et al., 2018b). While each of these measures is tightly correlated with chronologic age, each clock has important distinguishing features. Pan-tissue age was developed across many cell types and tissues and is accelerated in disease states. Extrinsic age was constructed to be positively correlated with methylation-based estimates of senescent (and inversely correlated with naïve) cytotoxic T lymphocytes. Second-generation clocks, including Phenotypic and Grim Age, are strongly predictive of healthspan and lifespan. Skin & Blood Age was developed in fibroblasts, keratinocytes, buccal cells, endothelial cells, lymphoblastoid cells, skin, blood, and saliva, and is highly accurate in estimating chronologic age. In addition to these methylation age measures, we examined a methylation-based estimate of telomere length, DNAmTL, which effectively captures the replicative history of cells and is highly predictive of age-related pathologies (Lu et al., 2019b). Based on DNA methylation levels at 140 CpGs, this measure was developed in leukocytes and adipose tissue from 3,334 individuals from three large cohort studies and was found to strongly correlate with leukocyte telomere length (Lu et al., 2019b). It is more strongly inversely associated with chronologic age than leukocyte telomere length and outperforms leukocyte telomere length in predicting time to death (Lu et al., 2019b).

### Statistical Analysis

For each epigenetic clock, we calculated a rate-of-aging measure as the ratio of the difference between DNA methylation (DNAm) age at Visit 2 and Visit 1 to the difference between chronologic age for the same visits:

$$R=\frac{DNAm\;age_{visit\;2}-DNAm\;age_{visit\;1}}{chronologic\;age_{visit\;2}-chronologic\;age_{visit\;1}}.$$

In addition, we calculated a rate-of-shortening for DNAmTL, using the ratio of the difference in DNAmTL between Visit 2 and Visit 1 to the difference between chronologic age for the same visits. We compared the rate-of-aging for each clock and rate-of-shortening in DNAmTL between PLWH and uninfected controls using Welch’s *t*-test. Bivariate analyses using simple linear regression models examining associations between rate-of-aging for each epigenetic measure and each predictor variable of interest were performed without adjustment. Multivariate linear regression was used to examine the association between rate-of-aging (dependent variable) and HIV status, with models adjusted for additional covariates, including baseline DNAm age or estimated DNAmTL, race (white *vs.* non-white), ethnicity (Hispanic *vs.* non-Hispanic), body mass index, cumulative pack-years of smoking tobacco, current Hepatitis B surface Antigen status, absoluate CD4 T cell count (cells/mm^3^), and cumulative HIV viral load (defined as the average HIV plasma viral load [copies/mL] between two consecutive viral load measurements, multiplied by the number of years between the two measurements [viremia copy years], which are summed over a specified time interval to calculate the area-under-the-curve (AUC)) (Wang et al., 2018). The AUC values were then log10-transformed and evaluated at each time interval. For cumulative viral load at Visit 1, we calculated from the first post HIV seroconversion visit with a reported HIV viral load up to and including the viral load at Visit 1. For cumulative viral load at Visit 2, we used plasma viral loads from visits ranging from the first visit with a reported HIV viral load up to and including the viral load at Visit 2.

## Results

Table 1 reports our study population’s demographic and clinical characteristics at the baseline visit (Visit 1). Participants were aged 23–72 years and were followed for an average of 6.7 years between Visit 1 and Visit 2 (range 0.3–19.6 years). By design, HIV-infected and uninfected groups were age-matched and had similar times between visits. Most men in both HIV-infected and uninfected groups were white and non-Hispanic. There were more non-white men among the uninfected group (26 *vs.* 9, *p* = 0.0026), consistent with the larger MACS biomarker study from which these participants were drawn. Both groups had similar numbers of participants who had ever smoked (57 *vs.* 54); HIV-infected men had a slightly higher mean cumulative pack-years of tobacco smoking than uninfected controls (12.5 *vs.* 8.6 pack-years) but the difference was not statistically significant (*p*-value = 0.082). There were few participants in both groups (one HIV-infected and two uninfected) who were positive for Hepatitis B surface antigen.

**TABLE 1:** Baseline characteristics of study participants.

Characteristic	HIV infected (N = 101)	Uninfected (N = 100)	*p-value*
Age (years), Mean ± SD	37.2 ± 7.6	37.9 ± 7.9	*0.50*
Ethnicity (non-hispanic), No. (%)	95 (94)	92 (92)	*0.77*
Race (white), No. (%)	92 (91)	74 (74)	*0.0026*
History of tobacco smoking (cumulative pack-years), Mean ± SD	12.5 ± 16.4	8.6 ± 14.4	*0.082*
Ever smoker, No. (%)	59 (58)	54 (54)	*0.62*
Body mass index (kg/m^2^), Mean ± SD	24.9 ± 3.6	25.3 ± 4.1	*0.48*
Hepatitis B surface antigen-positive, No. (%)	1 (1)	2 (2)	*0.99*
Time between visits (years), Mean ± SD	7.0 ± 3.5	6.5 ± 4.3	*0.31*
Cumulative plasma HIV Viral Load (log 10 viremia copy years) at Visit 1^a^, Mean ± SD	4.6 ± 0.8	N/A	N/A
Cumulative plasma HIV Viral Load (log 10 viremia copy years) at Visit 2^b^, Mean ± SD	5.3 ± 0.6	N/A	N/A
Absolute CD4 T cell count (cells/mm^3^) at Visit 1, Mean ± SD	825 ± 365	N/A	N/A
Absolute CD4 T cell count (cells/mm^3^) at Visit 2, Mean ± SD	702 ± 435	N/A	N/A

a Cumulative viral load calculated from first reported HIV viral load after documented HIV seroconversion up to and including Visit 1.

b Cumulative viral load calculated from first reported HIV viral load after documented HIV seroconversion up to and including Visit 2.

### PLWH Exhibit Faster Rate-of-Aging in Pan-Tissue, Extrinsic, and Phenotypic Clocks and Faster Rate-of-Shortening of DNAmTL Than Uninfected Controls

**FIGURE 1:**
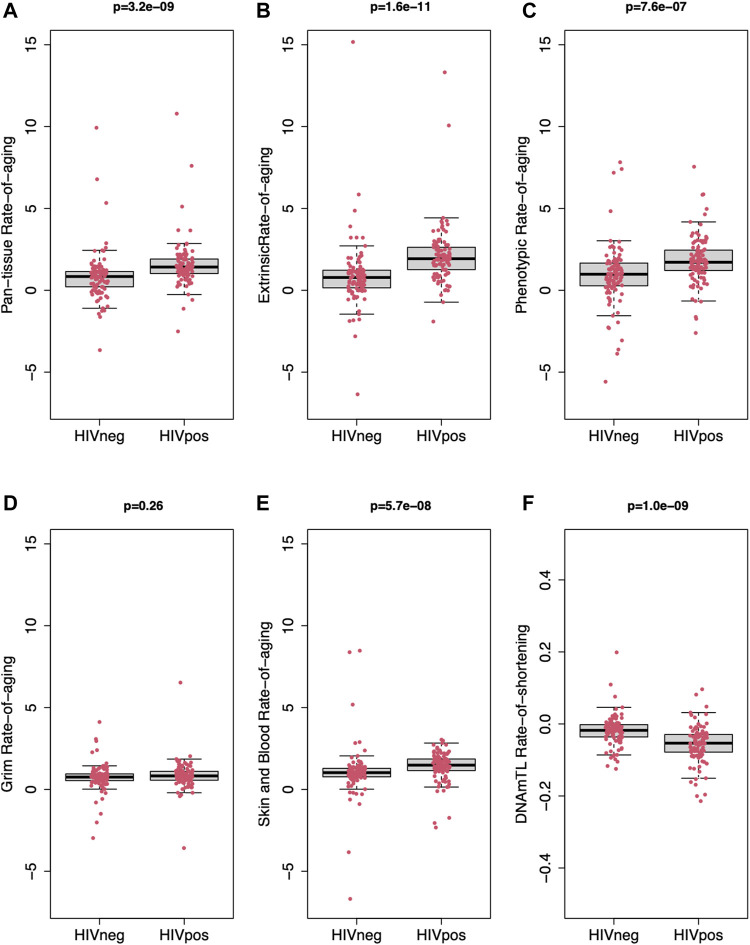
Full distributions for rate-of-aging in peripheral blood mononuclear cells of HIV-infected participants (HIV POS, *n* = 101) and uninfected controls (HIV NEG, *n* = 100). Rate-of-aging, expressed as the ratio of (change in DNAm age)/(change in chronologic age), for each individual from Visit 1 to Visit 2, is shown on the y-axis for the Pan-tissue clock [Panel **(A)**], Extrinsic clock [Panel **(B)**], Phenotypic clock [Panel **(C)**], Grim clock [Panel **(D)**], and the Skin & Blood clock [Panel **(E)**]. Rate-of-shortening in a methylation-based estimate of telomere length, DNAmTL, expressed as (change in DNAmTL)/(change in chronologic age) from Visit 1 to Visit 2, is shown in Panel **(F)**. The heavy black line indicates the median value; boxes represent 25th (Q1) to 75th (Q3) percentile, whiskers represent 5th–95th percentiles. *p* values shown are for the comparison between HIV-infected and uninfected men (Welch’s *t*-test). The y-axis of Panel F has units of relative units per year of chronologic age, and is accordingly on a different scale from that of the other panels.

Figure 1 reveals the distribution of rate-of-aging outcomes for HIV-infected and uninfected participants for each clock and the estimated rate-of-shortening of DNAmTL. The average rate-of-aging was significantly higher in HIV-infected compared with uninfected men for the Pan-tissue clock (1.9 *vs.* 0.9 epigenetic years per year of chronologic age, Welch’s *t*-test *p* < 0.001), Extrinsic clock (2.1 *vs.* 0.9, *p* < 0.001), and Phenotypic clock (1.8 *vs.* 0.8, *p* < 0.001). These findings all demonstrate an epigenetic rate-of-aging in HIV-infected men that is approximately twice as fast as that of uninfected men. There was a marginal difference between HIV-infected and uninfected men in rate-of-aging in the Skin & Blood clock (1.4 *vs.* 1.1 years per year of chronologic age, *p* = 0.052), and no significant difference in rate-of-aging of the Grim clock (0.83 *vs.* 0.75, *p* = 0.50). The average rate-of-shortening in DNAmTL was significantly faster in HIV-infected men (−0.056 units per year of chronologic age *vs.* −0.019, *p* < 0.001), indicating a nearly 3-fold increase in rate-of-shortening compared with uninfected men.

**FIGURE 2:**
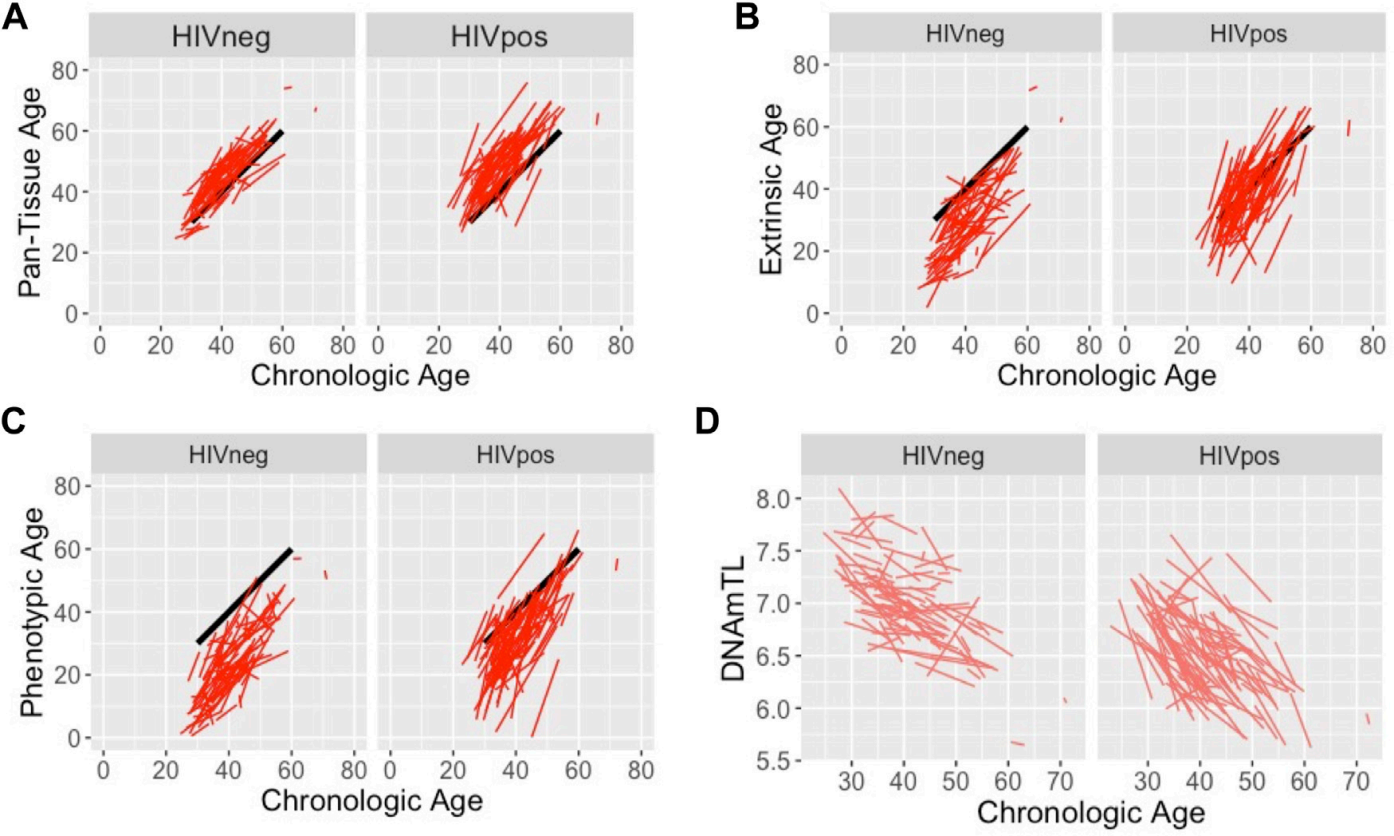
Individual trajectories from Visit 1 to Visit 2 for the Pan-Tissue, Extrinsic, and Phenotypic clocks, and estimated DNAmTL, in HIV-infected (HIVPOS), and uninfected (HIV NEG) men. Trajectories for each participant are plotted with DNAm age (years) or estimated DNAmTL (relative units) on the y-axis, and chronologic age (years) at each visit on the x-axis, for the Pan-Tissue [Panel **(A)**], Extrinsic [Panel **(B)**], and Phenotypic [Panel **(C)**] clocks, and estimated DNAmTL [Panel **(D)**]. Heavy black lines are added for reference to indicate a slope of 1 where the rate of epigenetic aging is equal to the rate of change in chronologic age.

Figure 2 reveals the individual trajectories for Pan-Tissue (2A), Extrinsic (2B), and Phenotypic (2C) Age for HIV-infected and uninfected men from Visit 1 to Visit 2. These figures illustrate the heterogeneity in epigenetic aging patterns across and within both groups, but also highlight higher starting points in methylation age at Visit 1 as well as steeper slopes from Visit 1 to Visit 2 in HIV-infected compared with uninfected men. While the steeper slopes correspond to the faster rate-of-aging shown in Figures 1A–C, these plots also demonstrate significantly higher starting points for methylation age at Visit 1 for Extrinsic age (mean 32.4 *vs.* 30.0 years in HIV-infected *vs.* uninfected, *p* = 0.0035), and Phenotypic age (mean 26.2 *vs.* 20.6 years in HIV-infected *vs.* uninfected, *p* < 0.001). The mean starting point of Pan-Tissue age was slightly higher in HIV-infected compared with uninfected men, but was not significant (43.9 *vs.* 42.3 years, *p* = 0.19). Figure 2D reveals the individual trajectories of DNAmTL in HIV-infected and uninfected men, demonstrating shorter starting DNAmTL at Visit 1 (mean 6.711 *vs.* 7.044 units in HIV-infected *vs.* uninfected, *p* < 0.0001), and the steeper (downward) slope/faster rate-of-shortening in DNAmTL in the HIV-infected group compared with uninfected controls consistent with Figure 1F. There were no significant differences in starting values in the Skin & Blood clock (39.8 *vs.* 38.3 years, *p* = 0.26), nor in starting values in the Grim clock (39.4 *vs.* 38.7 years, *p* = 0.57) among the HIV-infected and uninfected groups (Supplementary Figure S1).

### Factors Associated With Faster Epigenetic Rate-Of-Aging in Clocks and Rate-of-Shortening in DNAmTL

Results of our preliminary bivariate analyses examining associations between rate-of-aging and each predictor variable of interest are summarized in Supplementary Table S1. HIV status was very strongly associated with a significantly faster rate-of-aging in the Pan-Tissue, Extrinsic, and Phenotypic clocks, and a significantly faster rate-of-shortening in DNAmTL (all *p* ≤ 0.0006). Rate-of-aging in the Skin & Blood clock was marginally faster in HIV-infected men, with borderline significance (*p* = 0.052). Non-white race was associated with a slower rate-of-aging in the Extrinsic clock and a slower rate-of-shortening in DNAmTL (both *p* < 0.002). While there was a marginally higher cumulative history of tobacco smoking in HIV-infected individuals (Table 1), rate-of-aging in each clock and rate-of-shortening in DNAmTL was not associated with tobacco smoking. Among only HIV-infected men, lower absolute CD4 T cell count at Visit 1 was modestly associated with a faster rate-of-aging in the Extrinsic clock (*p* = 0.025), and faster rate-of-shortening in DNAmTL (*p* = 0.011). Lower absolute CD4 T cell count at Visit 2 was significantly associated with faster rate-of-aging in the Pan-Tissue, Extrinsic, and Phenotypic clocks, and a faster rate-of-shortening in DNAmTL (all *p* < 0.002), but was only marginally associated with rate-of-aging in the Skin & Blood clock (*p* = 0.056). Starting with a lower baseline DNAm age was associated with a faster rate-of-aging in the Phenotypic and Skin & Blood clocks, while starting with a shorter DNAmTL was marginally associated with a faster rate-of-shortening of DNAmTL.

Results from multivariate regression models examining factors associated with rate-of-aging in each epigenetic clock, and rate-of-shortening in DNAmTL, are shown in Table 2. In multivariate models adjusted for baseline DNAm age or DNAmTL, race, ethnicity, tobacco smoking, BMI, Hepatitis B status, and HIV status, HIV status remained very strongly associated with a faster rate-of-aging in Pan-Tissue, Extrinsic, and Phenotypic clocks, and a faster rate-of-shortening of DNAmTL (all *p* < 0.0009). Although the associations tended to be weaker, non-white race was still significantly associated with slower rate-of-aging in the Extrinsic clock and rate-of-shortening of DNAmTL, and lower baseline DNAm age was significantly associated with faster rate-of-aging in the Phenotypic and Skin & Blood clocks. Tobacco smoking continued to have no significant associations with rate-of-aging in any clock or with rate-of-shortening in DNAmTL. In multivariate analyses including only HIV-infected men, models which adjusted for all of the same covariates (except HIV status) plus cumulative HIV viral load and absolute CD4 T cell count showed that higher cumulative viral load at either Visit 1 or Visit 2 was associated with a faster rate-of-aging only in the Grim clock. When taking all of the other factors into account, absolute CD4T cell counts at either Visit 1 or Visit 2 no longer had statistically-significant associations with the rates of any of the epigenetic measures. There were only marginal associations (*p* = 0.07) between lower absolute CD4 T cell count at Visit 1 and slower rate-of-aging in the Grim clock, and lower absolute CD4 count at Visit 2 and faster rate-of-shortening in DNAmTL.

**TABLE 2:** Factors associated with rate-of-aging for each epigenetic clock: results from multivariate analysis.

	Rate-of-aging											
	Pan-tissue clock		Extrinsic clock		Phenotypic clock		Grim clock		Skin & blood clock		DNAmTL	
	β	p	β	p	β	p	β	p	β	p	β	p
Baseline DNAm age (by clock) or estimated TL (by DNAmTL)	−0.025	0.082	−0.015	0.27	−0.045	**5.1x10** ^**−4**^	−0.0032	0.75	−0.029	**0.0084**	−7.4 × 10^−3^	0.48
Race, non-white	0.24	0.45	−0.89	**0.034**	0.35	0.37	−0.22	0.23	−0.45	0.095	0.025	**0.016**
Ethnicity, hispanic	0.43	0.37	−0.46	0.46	0.40	0.49	−0.11	0.70	0.67	0.099	−0.013	0.40
History of tobacco smoking, cumulative pack-years	0.0080	0.30	0.0014	0.89	0.0071	0.45	0.0017	0.73	0.0079	0.22	−6.0 × 10^−5^	0.80
Body mass index, kg/m^2^	−0.012	0.69	−0.0042	0.91	0.017	0.62	0.0015	0.93	−0.0095	0.70	−0.0011	0.23
Hepatitis B sAg positive	0.28	0.76	−0.82	0.48	−0.44	0.69	−0.23	0.65	−0.029	0.97	−0.0074	0.80
HIV-infected^a^	0.79	**8.8** × **10** ^**−4**^	1.1	**3.3** × **10** ^**−4**^	1.29	**8.5** × **10** ^**−6**^	0.037	0.78	0.28	0.15	−0.035	**1.4** × **10** ^**−5**^
Cumulative plasma HIV viral load, visit 1^b^, log 10 viremia copy years	0.27	0.36	0.33	0.37	0.11	0.70	0.40	**0.018**	−0.065	0.65	−0.0096	0.32
Cumulative plasma HIV viral load, visit 2^c^, log 10 viremia copy years	0.092	0.79	−0.074	0.86	−0.10	0.77	0.42	**0.035**	−0.11	0.52	−0.0089	0.43
Absolute CD4 T cell count, visit 1^b^, cells/mm^3^	−9.0 × 10^−4^	0.35	−4.0 × 10^−4^	0.73	−9.0 × 10^−4^	0.31	1.0 × 10^−3^	0.070	−2.6 × 10^−4^	0.58	−1.9 × 10^−5^	0.51
Absolute CD4 T cell count, visit 2^c^, cells/mm^3^	−5.9 × 10^−4^	0.44	−8.5 × 10^−4^	0.35	−7.6 × 10^−4^	0.31	4.7 × 10^−4^	0.28	−4.8 × 10^−4^	0.19	4.4 × 10^−5^	0.070

a Multivariate models adjusted for baseline DNAm age or estimated TL, race, ethnicity, tobacco, BMI, hepatitis B status, and HIV status, including all individuals. Significant p-values are shown in bold.

b Models adjusted for baseline DNAm age or estimated TL, race, ethnicity, tobacco smoking, BMI, hepatitis B status, plus cumulative HIV viral load at Visit 1, and CD4 count at Visit 1, including only HIV-infected individuals.

c Models adjusted for baseline DNAm age or estimated TL, race, ethnicity, tobacco smoking, BMI, hepatitis B status, plus cumulative viral load at Visit 2, and CD4 count at Visit 2, including only HIV-infected individuals.

## Discussion

This study is one of the first and largest longitudinal analyses in PLWH compared with age-matched controls that examines epigenetic aging at two time points in the same individuals. It is also one of the few longitudinal studies that begins its analyses shortly after documented HIV infection in PLWH. Key findings from our study include the observation that living with untreated HIV infection leads to an epigenetic rate-of-aging in peripheral blood that is approximately twice as fast as that of uninfected populations and leads to a rate-of-shortening in DNAmTL that is approximately 3-fold faster than controls. These findings emphasize the critical impact of HIV on biologic aging, not only in elevating DNAm age above that of uninfected controls but also in increasing the rate-of-aging over the years following initial HIV infection.

Our observation of the impact of HIV on rate-of-change in epigenetic age is consistent with a recent study demonstrating an increase in the rate of epigenetic aging over time in youth with perinatally acquired HIV (Shiau et al., 2021). Comparing 32 youth with perinatally acquired HIV and 8 youth who were perinatally exposed without infection, the authors found significantly higher rate-of-aging in the Pan-tissue clock (1.2 years per year of chronologic age in infected *vs.* 1.0 in exposed youths, using data from two time points. In addition, they found that higher viral load, as quantified by the area under the curve, was associated with an increase in rate-of-aging in Pan-tissue age. While we have recently shown an early and substantial impact of HIV on the epigenetic aging process in a study comparing peripheral blood epigenetic ages in MACS participants pre- and post-HIV seroconversion with age-matched controls (Breen et al. *submitted/under revision*), the recent findings from the work of Shiau et al. (2021) and our current study suggest ongoing cellular and epigenetic changes post-seroconversion that evolve and persist over the duration of the years following infection. Significantly, we further demonstrate that the relationship between HIV and epigenetic rate-of-aging is consistent across three epigenetic clocks. We find that the effect of HIV on rate-of-shortening of DNAmTL is even more pronounced, suggesting an important impact of untreated HIV on cellular replicative history.

Limitations of our study include the lack of additional time points across the years of infection to examine for non-linearities in the relationship between HIV infection and epigenetic aging with longer duration of infection. Future studies are needed to examine 3 or more visits within HIV-infected individuals over the years following infection. We utilized the MACS cohort because of its wealth of archival longitudinal specimens from the pre-HAART era, with carefully annotated data on immunologic, virologic, and clinical measures for each participant. A limitation of our study is that there were no women in this cohort, and there were very small numbers of non-white participants. Future studies are needed to examine the relationship between HIV status and rate-of-aging in large longitudinal cohorts that include women and representation from diverse racial and ethnic groups. Finally, because this study focuses on epigenetic aging prior to highly active anti-retroviral therapy (HAART), our findings are not directly translatable to PLWH who have initiated HAART. Additional studies are in progress to examine the pace of epigenetic aging in PLWH while on therapy.

This work emphasizes the important relationship between HIV infection and epigenetic aging, not only in elevating the estimated DNA methylation age across clocks, but also having a significant effect on the rate-of-change in multiple clocks as well as on an estimate of cell replicative history. Because these clocks are closely tied to chronologic age and healthspan and lifespan, the acceleration of aging may represent a mechanism linking HIV and earlier onset of age-related illnesses. Future research is needed to investigate targeted therapies to prevent accelerated cellular senescence and functional decline in an aging population of PLWH.

## Data Availability

The raw Infinium MethylationEPIC BeadChip methylation data that support the findings of this paper will be available from the MACS/WIHS Combined Cohort Study (MWCCS) but restrictions apply to the availability of these epigenome-wide data. Per MWCCS policies, these raw data will not be released until the original aims of our approved study are complete. When available, MWCCS data are accessible through a concept sheet approval process, as described in: https://statepi.jhsph.edu/mwccs/work-with-us/. The MWCCS concept sheet number for all data related to this paper is C15039. All calculated epigenetic clock and estimated telomere length data utilized in our analyses, as well as necessary deidentified demographic or descriptive data, are provided in the supplemental Excel file.

## Supplementary Material

Supplementary Figure 1 Individual trajectories of Skin & Blood and Grim clocks in HIV-infected and uninfected men. Trajectories for each participant are plotted with DNAm age (years) on the y-axis, and chronologic age (years) at each visit on the x-axis, for the Grim [Panel **(A)**] and Skin & Blood [Panel **(B)**] clocks. Heavy black lines indicate a slope of 1 where the rate of epigenetic aging is equal to the rate of change in chronologic age.
