# Supplementary material for: Increased Rate of Epigenetic Aging in Men Living With HIV Prior to Treatment
Source: Front Genet. 2022 Feb 28;12:796547. doi: 10.3389/fgene.2021.796547 (PMC8919029; doi:10.3389/fgene.2021.796547)
Supplement: Supplementary file 1 [file Table1.docx]

**Supplemental Table 1. Bivariate analyses* examining factors associated with rate-of-aging for each epigenetic clock.**

|  | **Rate-of-aging** | | | | | | | | | | | |
| --- | --- | --- | --- | --- | --- | --- | --- | --- | --- | --- | --- | --- |
|  | **Pan-tissue clock** | | **Extrinsic clock** | | **Phenotypic clock** | | **Grim clock** | | **Skin & Blood clock** | | **DNAmTL** | |
|  | **β** | **p** | **β** | **p** | **β** | **p** | **β** | **p** | **β** | **p** | **β** | **p** |
| Baseline DNAm age (by clock) or estimated TL (by DNAmTL) | -0.019 | 0.13 | -0.0026 | 0.84 | -0.031 | **0.0061** | -0.0013 | 0.80 | -0.025 | **0.011** | 0.017 | 0.062 |
| Race, non-white | 0.015 | 0.96 | -1.18 | **0.0015** | 0.12 | 0.73 | -0.26 | 0.098 | -0.34 | 0.15 | 0.031 | **0.0011** |
| Ethnicity, Hispanic | 0.37 | 0.39 | -0.82 | 0.14 | 0.61 | 0.24 | -0.23 | 0.33 | 0.31 | 0.39 | 0.0050 | 0.72 |
| History of tobacco smoking,  cumulative pack-years | 0.0058 | 0.42 | 0.0058 | 0.54 | -0.0018 | 0.84 | 0.0022 | 0.59 | 0.0036 | 0.55 | -2.1x10^-4^ | 0.37 |
| Body mass index, kg/m^2^ | -0.026 | 0.37 | -0.023 | 0.54 | -0.016 | 0.66 | -9.8x10^-4^ | 0.95 | -0.025 | 0.29 | -7.1x10^-4^ | 0.46 |
| Hepatitis B sAg positive | -0.035 | 0.97 | -0.85 | 0.47 | -0.69 | 0.53 | -0.19 | 0.70 | -0.18 | 0.81 | -9.6x10^-3^ | 0.75 |
| HIV-infected | 0.73 | **6.0x10^-4^** | 1.2 | **1.2x10^-5^** | 1.0 | **1.4x10^-4^** | 0.080 | 0.50 | 0.35 | 0.052 | -0.037 | **7.6x10^-8^** |
| Cumulative Plasma HIV Viral Load,  Visit 1, log 10 viremia copy years | 0.24 | 0.31 | 1.8 | 0.78 | -0.14 | 0.54 | 0.19 | 0.18 | -0.19 | 0.17 | 2.3x10-4 | 0.98 |
| Cumulative Plasma HIV Viral Load,  Visit 2, log 10 viremia copy years | 0.14 | 0.58 | -0.016 | 0.96 | -0.26 | 0.29 | 0.22 | 0.13 | -0.14 | 0.34 | -9.6x10-3 | 0.27 |
| Absolute CD4 count at Visit 1, cell/mm3 | -5.4x10^-4^ | 0.079 | -8.7x10^-4^ | **0.025** | -8.8x10^-5^ | 0.80 | 9.3x10^-5^ | 0.56 | -6.7x10^-5^ | 0.77 | 2.3x10^-5^ | **0.011** |
| Absolute CD4 count at Visit 2, cells/mm3 | -8.3x10^-4^ | **7.9x10^-4^** | -1.1x10^-3^ | **4.1x10^-4^** | -9.4x10^-4^ | **0.0019** | -2.7x10^-5^ | 0.84 | -4.0x10^-4^ | 0.056 | 4.4x10^-5^ | **4.9x10^-8^** |

*Bivariate analyses from simple linear regression models (unadjusted) examining the association between each clock and each covariate. All individuals (HIV-infected and uninfected) were included in each analysis. Significant p-values are shown in bold.
