# Supplementary figures and images for: Increased Rate of Epigenetic Aging in Men Living With HIV Prior to Treatment
Source: Front Genet. 2022 Feb 28;12:796547. doi: 10.3389/fgene.2021.796547 (PMC8919029; doi:10.3389/fgene.2021.796547)

A

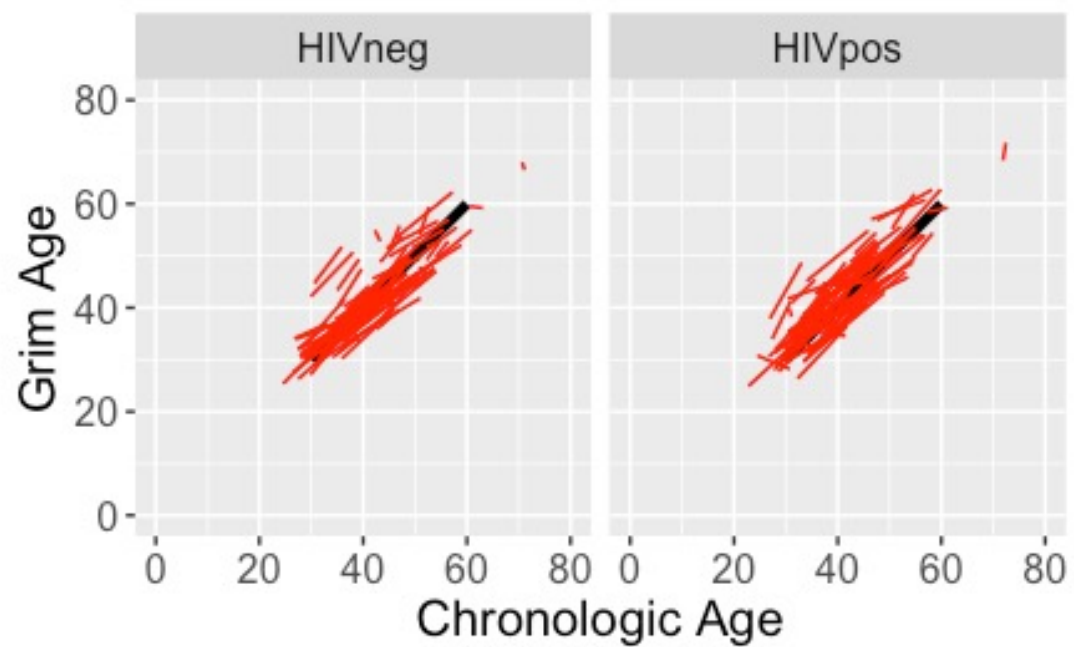

B

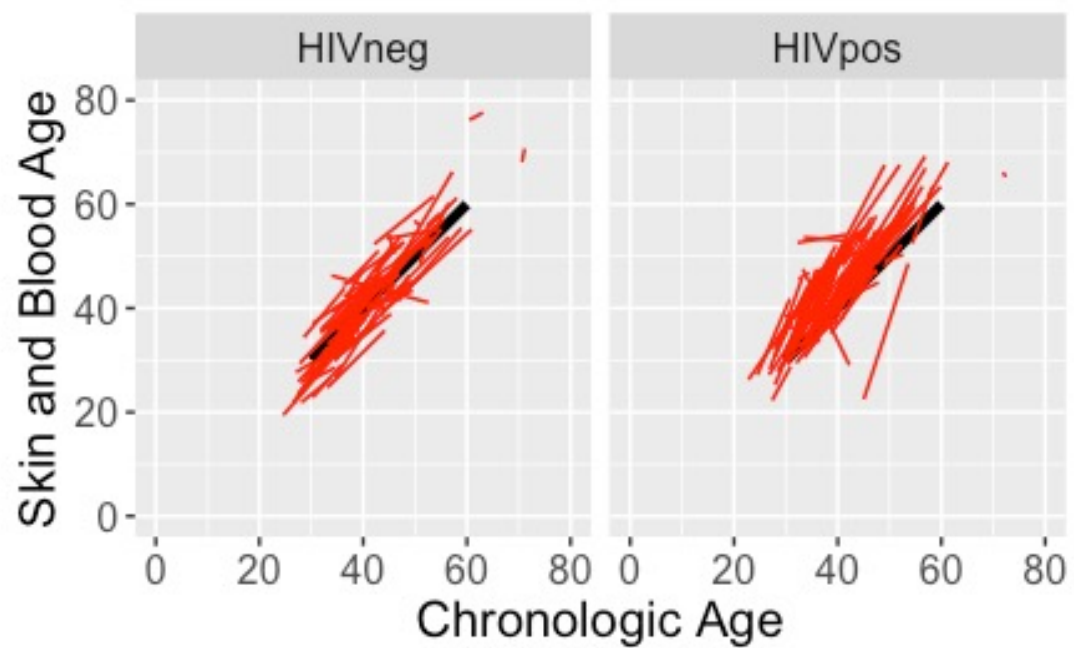

Supplement: Supplementary file 2 [file DataSheet1.PDF]
